# Organoid-on-a-chip (OrgOC): Advancing cystic fibrosis research

**DOI:** 10.1016/j.mtbio.2025.102148

**Published:** 2025-07-28

**Authors:** Minjie Zheng, Elisa Erice, Huiyi Wang, Lei Zhang, Charles H. Lawrie

**Affiliations:** a School of Microelectronics, Shanghai University, Shanghai, 201800, China; b Sino-Swiss Institute of Advanced Technology (SSIAT), Shanghai University, Shanghai, 201800, China; c Molecular Oncology Group, Biogipuzkoa Research Institute, Paseo Doctor Begiristain, San Sebastián, 20014, Spain

**Keywords:** Cystic fibrosis, Organ-on-a-chip, Organoid, Organoid-on-a-chip

## Abstract

Cystic fibrosis (CF) is an autosomal recessive disorder resulting from impaired anion transport in the epithelium of multiple organs, thereby affecting various physiological functions throughout the body. The heterogeneity of CF complicates drug development, highlighting the growing importance of individualized therapies. CF patient-derived organoid models and organ-on-a-chip (OOC) platforms are promising in vitro models for recapitulating CF pathology, owing to their high simulation fidelity, individualized therapeutic capabilities, cost-effectiveness, and high-throughput screening potential. This review systematically summarizes the technological development pathways of patient-derived organoids and OOC platforms for CF, along with recent advances in their applications to CF-related basic research, and particularly focuses on exploratory studies using organoid-on-a-chip (OrgOC) systems to elucidate CF pathogenesis and assess therapeutic approaches.

## Graphical abstract

**Figure:**
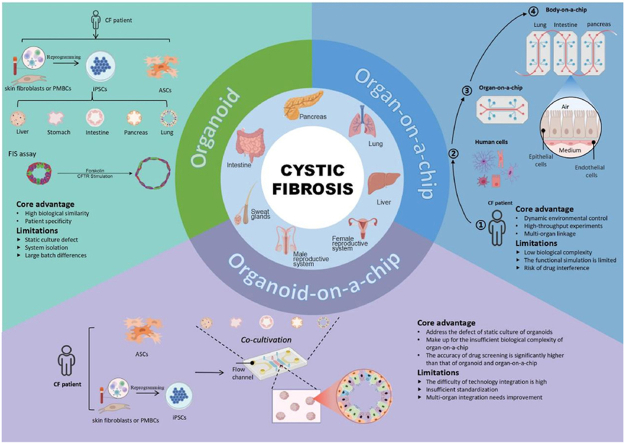

## 1 Introduction

### 1.1 Cystic fibrosis (CF)

**Fig. 1:**
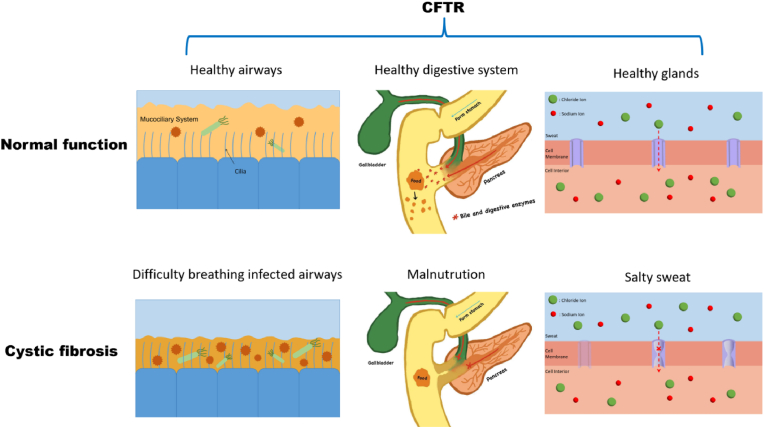
**A comparison is made between patients with cystic fibrosis and healthy individuals in the airways, digestive system, and sweat glands.** Ion transport dysfunction, caused by mutations in the CFTR gene, leads to abnormal mucus accumulation in the Vater juxtaglomerular region of the airway and digestive systems, resulting in progressive mucus obstruction and secondary infections. This dysfunction also impairs the delivery of digestive enzymes, such as bile and pancreatic enzymes, to the small intestine, ultimately causing progressive nutrient malabsorption and metabolic disorders. In the sweat glands, functional CFTR proteins regulate electrolyte balance by facilitating chloride ion transport across membranes. However, defective CFTR proteins cause chloride ions to accumulate in sweat, where they combine with sodium ions to form a high concentration of salts, leading to the characteristic hypertonic, salty-smelling sweat seen in affected individuals.

It is estimated that more than one hundred thousand people in 94 different countries suffer from cystic fibrosis (CF) [1]. CF is a genetic disorder affecting the respiratory, digestive, and reproductive systems, first described in 1938 by Andersen. In 1989, Collins and Tsui identified the underlying cause of CF as a mutation in the *CFTR* gene (Cystic Fibrosis Transmembrane Conductance Regulator) [2,3]. The CFTR protein regulates chloride and sodium ion movement across cell membranes and is present in various organs and tissues [4]. Mutations in the *CFTR* gene result in abnormally thick and viscous mucus in the lungs, pancreas, and other organs, hindering ciliary function and leading to recurrent lung infections [5], airway obstruction, and respiratory difficulties [6]. CF can also lead to additional complications, including inflammation, nutrient malabsorption, alterations in sweat gland function, and reproductive system changes (males primarily exhibit vas deferens lesions, while females are more commonly affected by cervical mucus thickening and metabolic disorders), among others (Fig. 1) [7]. The mutations in the *CFTR* gene are diverse and complex, with over 2000 distinct variants identified to date. These mutations can be categorized into seven major classes based on their molecular mechanisms and effects on protein function, as shown in Table 1 [8,9].

**Table 1:** Classification of CFTR mutations.

**Class**	Mutation Types	CFTR Defect	Mechanism
**Class I**	Nonsense, Frameshift, Splicing	Protein synthesis defect	mRNA premature termination, resulting in complete absence of CFTR protein (nonsense-mediated mRNA decay).
**Class II**	Missense, In-frame deletions	Protein processing and transport defects	Proteins are misfolded in the endoplasmic reticulum and are degraded by the ubiquitin-proteasome system, unable to reach the cell membrane.
**Class III**	Gating mutations	Channel gate defect	The CFTR protein can reach the cell membrane, but abnormalities in ATP binding or hydrolysis in the nucleotide-binding domain (NBD) result in the chloride ion channel being unable to open normally.
**Class IV**	Conductance defects	Conductivity of the channel decreases	The channel is open, but the permeability to chloride ions has significantly decreased.
**Class V**	Splicing/promoter mutations	Protein expression decrease	Abnormal mRNA splicing or reduced transcription efficiency leads to a decrease in functional CFTR protein.
**Class VI**	Stability defects	Protein stability decreases	Accelerated degradation of membrane-localized CFTR

Traditional treatment for CF patients have relied on digestive enzymes [10] and vitamin supplements for malnutrition [11], antibiotics for infections [12,13], and mucoactive agents for mucus thinning [14,15]. However, all these methods address only the symptoms, rather than the underlying cause of the disease [16,17]. The emergence of CFTR modulators—such as potentiators, correctors, read-through agents, amplifiers, and stabilizers—has led to a fundamental shift in CF treatments [18,19], with nearly 90 % of patients benefiting from CFTR modulator therapy [[20], [21], [22]]. However, despite these significant improvements, CFTR modulators are costly, require concomitant therapies, and necessitate long-term treatment [23,24]. Furthermore, for a subset of CF patients harboring specific CFTR variants, such as class 1 nonsense variants [25], no effective treatment exists. Moreover, some patients exhibit varying therapeutic responses to the same CFTR modulator drugs, despite sharing the same CFTR mutation [[26], [27], [28]], suggesting that the underlying pathological mechanisms of CF are not fully understood. This highlights the need for a more personalized treatment approach, considering both the genetic makeup of the patient and their specific CFTR variant [29,30]. However, this approach remains challenging, particularly in terms of selecting appropriate in vivo and in vitro models for screening, as these often fail to accurately replicate the disease state or the biological profile of the patient.

Over the past three decades, researchers have developed in vitro models of CF, including animal models and immortalized epithelial cell lines [[31], [32], [33]]. While animal models of CF offer valuable insights into the disease's pathophysiology, the process is both time-consuming and costly. Moreover, these models are non-human and fail to represent the full spectrum of disease manifestations in individual patients, nor do they accurately depict patient-specific disease phenotypes [34]. In contrast, immortalized epithelial cell lines derived from patients have made significant contributions to CF research [33]. However, acquiring these cell lines from patients remains inefficient, as only a few clones can be successfully expanded and maintained through multiple passages, requiring extensive adaptation and rigorous selection under in vitro two-dimensional culture conditions [35].

### 1.2 Insight into organoid, organ-on-a-chip and organoid-on-a-chip

#### 1.2.1 Organoid

In 2009, Clevers first introduced the concept of the organoid, a 3D tissue model formed through in vitro cell self-organization, capable of mimicking both the structure and function of in situ tissues [36]. In comparison to traditional (2D) cell cultures, organoids are cultured in a three-dimensional system that more closely resembles the human body, more accurately replicating tissue structure, metabolic function, and gene and protein expression of the original organ [37]. Organoid models can replicate the in vivo environment, preserve tumor heterogeneity, and play an increasingly pivotal role in the fields of personalized and precision medicine, including in CF research [38,39].

Traditional methods for organoid formation, based on the three-dimensional (3D) culture of mammalian stem cells with added growth factors, have several limitations. Although conceptually straightforward, these methods are challenging in practice, as it is difficult to precisely control both organoid development and the local environment. Additionally, they fail to replicate the complex, dynamic microenvironment found in developing organs [40]. In contrast, Organ-on-Chip (OOC) technology offers a promising approach to overcome these challenges.

#### 1.2.2 Organ-on-a-chip (OOC)

Organ-on-a-chip (OOC) technology integrates microfluidic channels with engineered biological tissue models to replicate key organ functions, offering a compact lab-on-a-chip system that enables the study of physiological processes in a controlled environment. This innovation has progressed to the point where multiple organ chips can be integrated to simulate systemic interactions, providing a more accurate and efficient platform for drug research and toxicity studies, thereby potentially reducing the reliance on traditional animal testing. In addition to providing physical stimulation, OOC technology features a perfusion-based culture system, enabling controlled delivery of nutrients and factors in the medium, precise regulation of concentrations, and a continuous supply of fresh medium and essential gases (such as oxygen) to the cells, while facilitating the removal of waste products. These features are essential for maintaining cell viability and function over extended periods. Furthermore, solute gradients can be generated in the flow, which is valuable for studying and inducing various cellular behaviors, including proliferation, migration, differentiation, inflammation, and tumor formation [41].

#### 1.2.3 Organoid-on-chip (OrgOC)

By combining the advantages of organoids and OOC, the organoid-on-a-chip (OrgOC) platform provides significant benefits in several key aspects [42,43]. First, microfluidic technology allows precise control of the microenvironment, providing highly controllable culture conditions that better simulate real physiological environments [44]. Second, the OrgOC platform, incorporating the latest advancements in OOC technology, can model the crosstalk between multiple tissues, revealing complex signaling and metabolic interactions between organs [45]. Additionally, the microfluidic system reduces heterogeneity in organoid cultures, thereby improving the accuracy and reproducibility of experimental data. The platform also supports high-throughput experimental designs, enabling parallel testing of multiple experiments, thereby accelerating drug screening and disease research [46]. 10.13039/100014337Furthermore, OrgOC can simulate various physiological parameters, including mechanical forces, temperature, and pressure, further enhancing its application in biomedical research. Finally, real-time monitoring and data acquisition enable dynamic observation of organoid growth and development, providing deeper insights into scientific investigations [47,48].

CF, a monogenic genetic disorder, has long faced a fundamental challenge in therapeutic development: the disconnect between preclinical models and clinical outcomes. While conventional 2D cell cultures can partially reflect CFTR functional abnormalities, they fail to replicate the 3D structure of the airway epithelium (e.g., spatial distribution of basal cells, goblet cells, and ciliated cells), mucus secretion dynamics (e.g., regulation of mucus layer thickness and viscosity), or transcellular signaling networks (e.g., the synergistic interaction between CFTR and the epithelial sodium channel). As a result, in vitro drug response evaluations often exhibit significant discrepancies from the therapeutic efficacy observed in patients. CF mouse models, owing to interspecies differences (e.g., airway mucus is primarily composed of mucin MUC5B rather than human MUC5AC), fail to recapitulate typical human CF phenotypes, such as chronic *Pseudomonas aeruginosa* infection and progressive pulmonary fibrosis [49].

Organoids, generated through the differentiation and culture of patient-derived pluripotent stem cells (iPSCs) or primary cells, retain the 3D structure of the airway epithelium, cellular heterogeneity, and mucus secretion function, thus more accurately reflecting the pathological characteristics of CF patients [50]. Meanwhile, Organs-on-Chip (OoC) platforms, leveraging microfluidic technology, can replicate physiological levels of fluid shear stress and mucus flow, dynamically regulate oxygen gradients [51], and further recapitulate the pathological microenvironment of CF airways (e.g., mucus retention and bacterial colonization) [52,53]. OrgOC models, based on organoid-OoC systems, are expected to significantly enhance the clinical relevance of CF pathology research and drug screening.

This review aims to comprehensively summarize the latest progress of organoid and OOC technologies in CF, introduce the development of OrgOC, and discuss the potential of OrgOC in the field of CF research.

## 2 Organoid and organ-on-a-chip (OOC) for cystic fibrosis research

### 2.1 Organoid

Organoids are formed through the self-organization of stem cells, including human pluripotent stem cells (PSCs), embryonic stem cells (ESCs), adult stem cells (ASCs), and cancer cells [36,[54], [55], [56]]. Intestinal and respiratory tracts cells are the most common source of cells used for CF organoid development [57]. The primary strategies for developing organoids in CF research involve culturing patient stem cells, which are then induced to differentiate and form organoids [58,59], or reprogramming patient somatic cells, such as skin or blood cells, into induced pluripotent stem cells (iPSCs), which are subsequently differentiated to form organoids [60,61].

#### 2.1.1 Intestinal, nasal, airway, and iPSC models

**Fig. 2:**
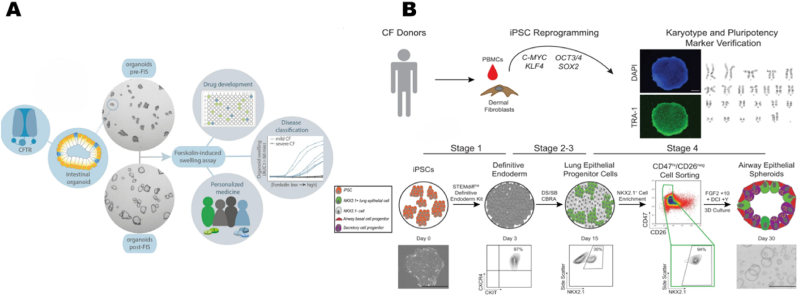
**Construction and application of cystic fibrosis organoids** (A): The forskolin-induced swelling (FIS) assay transforms molecular functional defects in CFTR into quantifiable morphological changes in organoids, such as swelling degree, serving as a key bridge that connects gene-protein-function-phenotype in CF research. It can be applied in functional assessment, drug screening, and individualized diagnosis and treatment. Copyright 2020, Elsevier. (B): Construction of airway organoids derived from CF patients. First, somatic cells were reprogrammed into induced pluripotent stem cells (iPSCs), and their pluripotency and G-band karyotype labeling (TRA-1 staining and DAPI nuclear labeling) were then tested. A schematic diagram of redirected differentiation to generate airway epithelial cell spheres was examined using flow cytometry to ensure differentiation efficiency. Copyright 2022, Springer Nature.

The first small intestinal organoids derived from adult stem cells were developed from mouse Lgr5+ stem cells [62], whereas human intestinal organoids are generated from intestinal stem cells (e.g., Crypt Base Columnar Cells (CBCs)) isolated from mucosal samples of the gastrointestinal tract, typically obtained through surgical biopsy. Rectal tissue is the most widely used tissue for personalized testing, as this tissue isolation process is less invasive, painless, suitable for all ages (including newborns and infants), and the samples are easily transportable to specialized laboratories for analysis [59]. Dekkers et al. were the first to establish an organoid model using rectal tissue from CF patients, finding that it did not respond to forskolin. However, responsiveness to forskolin was restored after treatment with CF transmembrane regulators or CRISPR-Cas9 correction of the mutation [63]. Based on this, researchers discovered that the forskolin-induced swelling assay (FIS) can accurately measure CFTR function (Fig. 2A) [59].

Human airway organoids (AOs), derived from adult stem cells in lung tissue, possess important features similar to those of human lung adult fine bronchioles [58,64]. AOs from CF patients can mimic *CFTR* mutations, and the consequent epithelial dysfunctions, such as increased mucus secretion [65]. Fresh samples are obtained through more invasive methods, such as bronchial brushing, alveolar lavage, and/or lung biopsy [57]. Sachs et al. developed human airway-like organoids that mimicked several key features of the human airway epithelium, including various cellular compositions, polarized pseudo-complex structures, and functional cells, such as multiciliated cells and mucus-secreting cells [58].

Reprogramming somatic cells into iPSCs for organoids developing significantly reduces the difficulty of sampling by utilizing various somatic cells. For example iPSCs derived from CF patient skin fibroblasts or PMBCs were differentiated into airway epithelial cells that both morphologically and functionally resembled native airway epithelial cells (Fig. 2B) [60]. Using these iPSC-derived airway organoids, the researchers conducted various studies, including evaluating the baseline function of CFTR, testing the efficacy of CFTR-modulating drugs, such as VX-770 and VX-809, and, analyzing the genetic background dependence of drug responses in different individuals. They also used these organoids to predict the potential responses of CF patients to specific drug treatments and assessed the stability of organoids after long-term culture and cryopreservation. Additionally, the use of experimental compounds, such as readthrough agents and NMD (nonsense-mediated decay) pathway inhibitors, was found to significantly improve CFTR function in patients with CFTR class 1 variants, which carry premature termination codons [60].

#### 2.1.2 Application of CF Organoid models

In cystic fibrosis (CF) research, the use of organoids has markedly improved our capacity to study the disease in a physiologically relevant context. Organoids are employed not only to verify CFTR function, assess the regulatory effects of drugs on CFTR, and evaluate drug responses, but also to analyze disease severity and predict prognosis. Moreover, organoids derived from individual patients enable the study of personalized drug responses, facilitating more precise treatment monitoring for CF patients (Table 2).

**Table 2:** Research application of patient-derived organoids in CF.

Application	Detailed Description	Organoid Type	Reference
CFTR Function Validation & Drug Evaluation	● Employing organoids to validate CFTR protein function and activity. ● Assessing the restorative effects of potential therapeutic agents on CFTR function. ● Investigating the penetration and pharmacodynamics of drugs in vivo. ● Identifying novel drug candidates through screening that can effectively enhance CFTR function.	Intestinal organoids	[38,63,[66], [67], [68], [69], [70], [71], [72], [73], [74]]
		Rectal organoids	[75,76]
		Acinar/Ductal organoids	[77]
		Airway organoids	[58]
		Lung organoids	[78,79]
		Nasal organoids	[[80], [81], [82]]
Disease Severity & Prognosis Assessment	● Assessing CF disease severity in patients by analyzing morphological features, cellular differentiation, and functional indicators in intestinal organoids. ● Monitoring inflammatory responses, mucus secretion, and bacterial infections in intestinal organoids to predict patient prognosis.	Intestinal organoids	[38,50,65,83]
Individualized Drug Response & Treatment Monitoring	● Investigating variations in drug response among patients using patient-derived organoids. ● Monitoring changes in organoids during treatment, including alterations in cellular structure, functional recovery, and inflammatory responses, to evaluate treatment efficacy and adjust therapeutic strategies.	Intestinal organoids	[[84], [85], [86], [87], [88], [89], [90], [91], [92], [93]]
CFTR Variant Identification & Treatment Strategy Development	● Investigating the functional characteristics and pathogenic mechanisms of various CFTR variants using organoid models. ● Evaluating the effectiveness of treatment strategies targeting specific CFTR variants, including gene editing and pharmacological therapy.	Intestinal organoids	[[94], [95], [96], [97], [98], [99], [100], [101], [102], [103], [104], [105], [106], [107], [108], [109], [110], [111], [112], [113]]
		Rectal organoids	[87,[114], [115], [116], [117]]
		Airway organoids	[112]
		Nasal organoids	[118]
Host-Pathogen Interaction Research	● Investigating the interactions between CF patients and pathogens using airway organoid models. ● Exploring the impact of pathogen infections on CFTR function and potential anti-infective treatment strategies.	Airway Organoids	[119,120]

### 2.2 Organ-on-a-chip (OOC) for cystic fibrosis research

The multi-system pathology of CF necessitates research models that can elucidate both organ-specific characteristics and systemic interactions. Currently, the lung-on-a-chip model offers a dynamic platform for analyzing mucus stasis, chronic infection, and inflammatory cascades by reconstructing the airway mucus-cilia barrier and CFTR-mediated ion transport. The pancreas-on-a-chip model focuses on pancreatic duct obstruction and enzyme secretion defects, highlighting the dual disruption of digestive and endocrine functions caused by CFTR mutations. An integrated, vascularized lung-on-a-chip model can simulate CF-associated vascular leakage and immune cell migration, thereby illustrating multi-organ interactions.

#### 2.2.1 Lung-on-a-chip

**Fig. 3:**
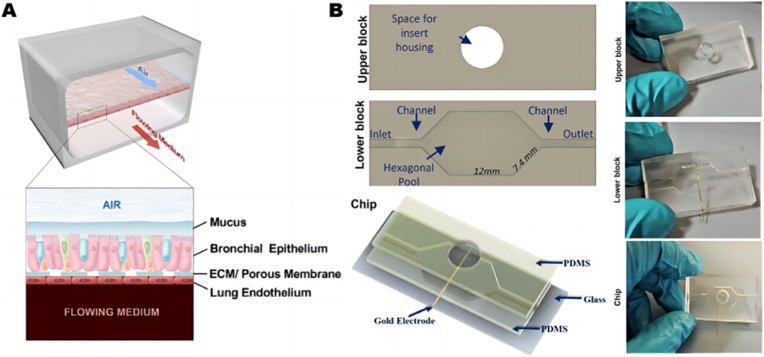
**lung-on-a-chip** (A) Diagram of the Airway Chip illustrating the pseudostratified bronchial epithelium cultured under an air-liquid interface (ALI) on the top surface of a porous membrane, which separates the upper epithelial channel from the lower vascular channel containing primary human lung microvascular endothelial cells adhered to the bottom of the same membrane and exposed to dynamic fluid flow. Reproduced with permission [122]. Copyright 2022, Elsevier. (B) Design of the microfluidic chip, featuring a top block with space for Transwell housing and a bottom block composed of a fluidic channel and a central hexagonal pool. A gold electrode was integrated into the device, and the chip was mounted on a glass slide for stabilization. Images of the top and bottom blocks of the device, as well as the bonded chip, are shown. Reproduced with permission [123]Copyright 2023, ACS Publications.

The lung-on-a-chip model is a crucial platform for studying respiratory diseases such as CF, as it can replicate the complex structure and function of lung tissues, including the alveolar-capillary interface [121]. Plebani et al. developed a microfluidic device that replicates the lung airways of CF patients (Fig. 3A) [122]. This chip was designed with dual microfluidic channels, featuring an extracellular matrix (ECM)-coated porous membrane with a 7-μm pore size, separating two parallel channels: the upper channel mimicking the airway epithelium and the lower channel mimicking the vasculature. The upper channel of the chip was seeded with primary human bronchial epithelial cells from CF patients or healthy donors, cultured under an air-liquid interface (ALI) to form a tightly packed monolayer. The lower channel was lined with lung microvascular endothelial cells, which were directly exposed to the flowing culture medium. The porous membrane surface of the chip is coated with ECM to promote cell attachment and growth. The dynamic fluid flow within the chip allowed it to replicate the physiological environment of the airways and blood vessels. Experimental results from the chip confirmed increased mucus accumulation, elevated cilia density, and accelerated cilia beating frequency in human CF airways. Furthermore, following inoculation with *Pseudomonas aeruginosa,* mucus in CF airways facilitated the growth of the pathogen, leading to increased secretion of inflammatory cytokines and aggregation of polymorphonuclear leukocytes (PMNs) in the airways.

Mazio et al. developed a reusable microfluidic chip for CF consisting of two PDMS blocks, with the upper layer providing a central space for Transwell insertion, and the bottom featuring fluidic channels and a central hexagonal culture cell [123]. Gold electrodes were integrated between the two PDMS blocks to enable real-time monitoring of electrophysiological properties of tissues, including trans-epithelial electrical resistance (TEER) and capacitance (Fig. 3B). Microrheology analysis (MPT) using fluorescently labeled nanoparticles was employed to assess the viscoelasticity and viscosity of mucus, while a fluorescent nanoparticle aerosol system was used to accurately measure the thickness of the mucus layer. The chip demonstrated electrical physiological differences between CF and non-CF epithelia, as well as a reduction in mucus thickness and viscosity following treatment with the CFTR modulator, VX-809.

#### 2.2.2 Pancreas-on-a-chip

**Fig. 4:**
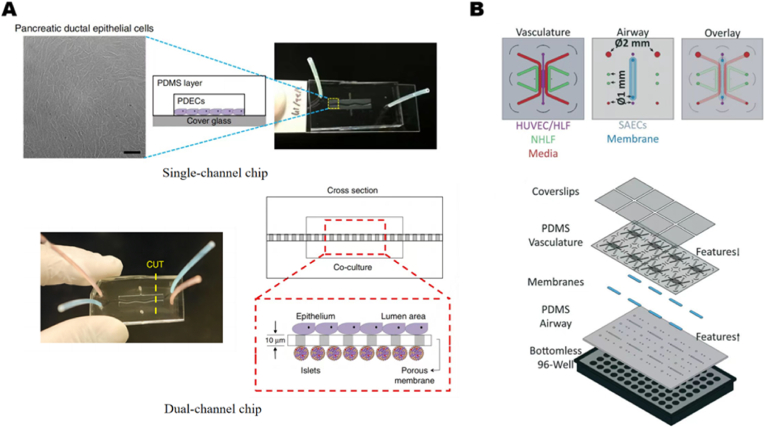
**Pancreas-on-a-chip, Microvascularized lung-on-a-chip** (A) The single-channel chip, which mimics the structure of pancreatic ducts with branches and progressively narrower diameters, and the inset shows pancreatic ductal epithelial cells (PDECs) cultured on the chip; and the dual-channel pancreatic chip, which consists of two cell culture chambers and a porous membrane in which PDECs and islet cells are cultured on each side of the porous membrane. Reproduced with permission [125]. Copyright 2019 Springer Nature. (B) Structural diagram of a 96-well format microvascularized human lung-on-a-chip: top coverslips, vascular layer, membrane, airway layer, and bottomless 96-well. The vascular layer consists of human umbilical vein endothelial cells (HUVECs) and human lung fibroblasts (HLF), as well as normal human lung fibroblasts (NHLF), cultured in separate channels. The airway layer consists of normal human small airway epithelial cells (SAECs) cultured in the channel under S-ALI conditions. Reproduced with permission [126] Copyright 2020, The Royal Society of Chemistry.

Pancreatic dysfunction plays a critical role in CF pathogenesis as the impaired CFTR protein in pancreatic ductal epithelial cells disrupts the secretion and transport of digestive enzymes resulting in exocrine pancreatic insufficiency (EPI) [124]. Furthermore, defects in the CFTR protein indirectly impair the endocrine function of pancreatic islets, potentially leading to cystic fibrosis-related diabetes mellitus (CFRD). This is caused by disrupted signaling between pancreatic ductal epithelial cells and pancreatic islet cells, which reduces insulin secretion. Mun et al. designed and fabricated two types of OOC devices (Fig. 4A). The first was a single-channel chip, combining a patterned PDMS block with a coverslip to mimic the structure of a pancreatic duct [125]. This chip cultured pancreatic ductal epithelial cells (PDECs) and monitored CFTR function using an iodide efflux assay. The second design was a dual-channel microchip composed of two patterned PDMS blocks with a thin porous membrane between them, serving as a separator and creating two culture channels that mimic the interactions between pancreatic ducts and pancreatic islet cells. The upper culture chamber is used to culture PDECs, while the lower chamber is used to culture islet cells. This design allows researchers to observe and analyze the effects of CFTR function on insulin secretion by islet cells. The use of both chips allowed the identification of CFTR's role in the direct regulation of insulin secretion for the first time and demonstrated the existence of a functional coupling between ductal cells and pancreatic islets.

#### 2.2.3 Microvascularized lung-on-a-chip

Microvascularization is critical for accurately replicating the physiological functions of organs, as it facilitates oxygen and nutrient delivery, metabolic waste removal, and the distribution of hormones and signaling molecules. In OOC models, microvascularization can emulate key aspects of lung microvasculature, including oxygen and nutrient exchange. This capability is particularly important for modeling CF, as impaired oxygen delivery is a hallmark of the disease, significantly affecting lung function. Mejías developed a microvascularized human lung OOC (Fig. 4B) [126], which includes a vascular layer fabricated via photolithography to mimic a microvascular network, a 3D-printed airway layer to model the respiratory tract, and an intermediate membrane layer constructed from PETE or collagen membranes to simulate the lung's basement membrane, facilitating gas and nutrient exchange. Using this platform, researchers demonstrated the role of CF airway epithelium in cytokine secretion by the innate immune system and in neutrophil recruitment.

## 3 Emerging organoid-on-a-chip (OrgOC) technology

As a fusion of organoid and microfluidic systems, OrgOC provides an innovative platform for biomedical research [127]. It integrates the structural and functional complexity of organoids with the controlled conditions of microfluidic chips, enabling precise simulations of both physiological and pathological processes (Table 3). Additionally, OrgOC holds significant potential for personalized medicine. By leveraging patient-derived cells, researchers can make precise predictions of individual drug responses, thereby supporting the development of personalized treatment plans. The initial approach for constructing organoid models involved culturing pluripotent or adult stem cells in a 3D environment. While conventional 3D culture techniques provide a straightforward and effective means of producing organoids in standard laboratory settings, their simplicity often compromises precise control. OrgOC utilize microfabrication techniques to construct bionic structures with microchannels and culture chambers, offering physical support for organoids. Through microfluidic systems, OrgOC dynamically regulate physiological microenvironment parameters, including fluid shear stress, nutrient/drug perfusion rate, and oxygen gradients. Simultaneously, organoids retain their 3D tissue structure and cellular heterogeneity, thereby replicating organ-level functional interactions and pathological processes in vitro, thus supporting disease mechanism research and drug response evaluation. Compared with traditional 3D-cultured organoids, the current OrgOC technology offers two main advantages: 1) it can provide an in vivo-like microenvironment, enhancing the accuracy of in vitro models; 2) it can promote organoid maturation and sustain their growth by co-culturing endothelial tissues. Achieving these advantages lays the foundation for constructing targeted OrgOC systems.

**Table 3:** Comparison of the characteristics of organoid, organ-on-a-chip and organoid-on-a-chip.

Dimension	Organoids	Organ-on-a-Chip	Organoid-on-a-Chip
**Definition**	Three-dimensional tissue models formed by self-organization of cells in vitro, miniature organ models that simulate the cell types, spatial structures, and partial functions of native organs.	Microfluidic-integrated miniature biochips simulate in vivo dynamic microenvironments through microchannel perfusion, enabling long-term cell/tissue culture and functional monitoring.	The integration technology of organoids and organ-on-a-chip provides a dynamic culture environment for organoids through microfluidic chips while preserving the biological complexity of organoids.
**Physiological Relevance**	**High** (3D self-organized structure, multi-cellular composition, and partial functional retention)	**Moderate** (dynamic microenvironment simulation, e.g., fluid shear stress and nutrient gradients, but with simplified monolayer/2D tissues)	**High (Integrating the biological complexity of organoids with the dynamic environment of organ-on-a-chip systems)**
**High-Throughput Screening**	**Low** (long culture cycles and high batch-to-batch variability, supporting only 10–20 samples per batch)	**High** (microfluidic design enables 40–100 independent channels for parallel drug/concentration testing)	**Moderate (limited by organoid culture efficiency, supporting 20**–**50 samples per chip, but 50 % more than organoids)**
**Manipulability**	**Low** (fragile structure complicates micro-operations like gene editing or localized drug delivery)	**High** (microfluidic channels allow precise control of flow rates and shear stress, enabling drug gradient delivery and localized stimulation)	**Moderate (combining the operational convenience of chips with the biological complexity of organoids)**
**Biobanking**	**High** (long-term cryopreservation of patient-derived organoids retains genetic background)	**Low** (relies on short-term cultured cell lines or primary cells, difficult to cryopreserve and recover original phenotypes)	**High (organoid cryopreservation adapted to chips)**
**Modeling Organogenesis**	**High** (simulates dynamic stem cell self-organization into organ primordia)	**Low** (primarily models mature organ microenvironments, rarely capturing developmental cell-cell interactions)	**High (organ-on-a-chip based dynamic environments support complete organoid development from stem cells to organ primordia)**
**Simulating Human Development and Disease**	**Moderate** (recapitulates single-organ developmental defects but lacks inter-organ signaling)	**Moderate** (supports multi-organ module connections but with limited biological complexity)	**High (integrates organoid developmental processes with chip-based multi-organ crosstalk, simulating dynamic pathologies)**
**Cost**	**Moderate** (primary costs from stem cell culture reagents; ∼$500–800 per sample)	**High** (high microfluidic chip fabrication (photolithography/PDMS molding) costs; ∼$1000–2000 per chip)	**High (combined costs of organoid culture and chip fabrication; ∼$1500**–**2500 per sample)**
**Ethical Considerations**	**Moderate** (involves ethical issues with embryonic stem cells (ESCs) or iPSCs, requiring ethics committee approval)	**Low** (primarily uses adult or immortalized cell lines, with minimal ethical concerns)	**Moderate** (similar to organoids)
**Live Imaging**	**Low** (opaque organoid matrices require fluorescent labeling or sectioning; long-term imaging damages tissues)	**High** (transparent chip materials (e.g., PDMS) enable real-time confocal imaging to monitor cell dynamics	**High (transparent chips support long-term, non-destructive 3D imaging of organoids)**
**Standardization**	**Low** (significant variability in culture conditions (matrix gel concentration, growth factors) Organoid structures/functions vary significantly across different laboratories)	**Moderate** (microfluidic design follows industry standards, but cell seeding density remains variable)	**Low** (lack of unified standards for the compatibility parameters between organoids and organ-on-a-chips (such as organoid size and perfusion rate)).

### 3.1 Microenvironmental control of organoids

#### 3.1.1 Mechanical forces

**Fig. 5:**
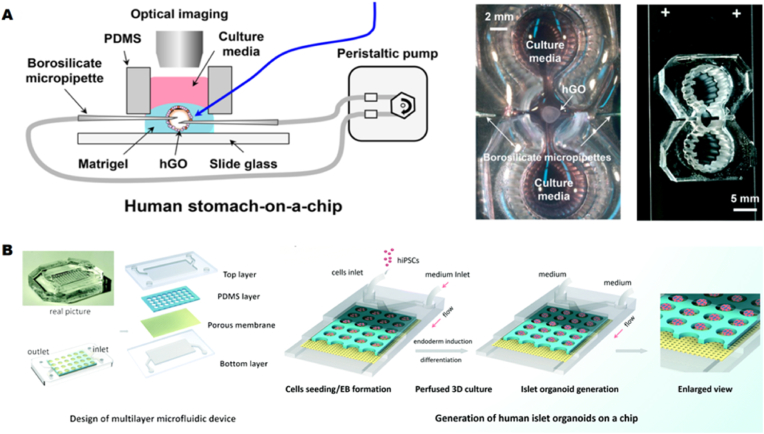
**Controlling the microenvironment of organoids-on-a-chip** (A) Schematic diagram illustrating the experimental setup for generating tubular flow in a peristaltic gastric organoid chip. The actual figure of the gastric organoid chip consists of an incubation chamber, two reservoirs, and connections between two micropipettes. Reproduced with permission [130] Copyright 2018, The Royal Society of Chemistry. (B) The islet organoid chip comprises four layers: the top layer, the through-hole PDMS layer, the porous membrane, and the bottom layer; the differentiation and generation process of islet organoids derived from human induced pluripotent stem cells (hiPSCs) on the chip under three-dimensional perfusion culture conditions. The islet organoid differentiation process on the chip under three-dimensional perfusion culture conditions includes embryoid body (EB) formation, endoderm induction, and islet organoid differentiation. Reproduced with permission [131] Copyright 2021 Wiley.

The developing embryo experiences various types of mechanical forces caused by the coordinated activity of large groups of cells (e.g., cardiac contractions, fetal respiratory movements) [128]. These forces work in conjunction with soluble morphogenetic factors and ECM signaling to regulate organ development and maturation [129]. Such critical forces are absent in conventional 3D culture environments. To address this limitation, recent studies have demonstrated mechanically actuatable microengineering platforms capable of generating and applying mechanical forces in organoids, akin to those in vivo. For example, Lee et al. developed a gastric microarray model composed of PDMS with three chambers: a central chamber for organoid culture and two adjacent chambers for storing and delivering culture medium (Fig. 5A) [130]. Gastric organoids derived from human hematopoietic stem cells were cultured in a matrix gel within the central chamber. Two borosilicate micropipettes were inserted into opposite sides of the organoids and connected to tubes running through a peristaltic pump, enabling bionic periodic pumping of fluid flow to mimic the luminal flow and rhythmic contractions of the stomach in vivo. The platform facilitated the robust growth of 3D human gastric organoids with controlled luminal flow. The cultivation of lung organoids for CF research also requires similar mechanical forces to replicate pulmonary movements, including contraction and expansion. Mechanical motion not only affects the organoid's response and sensitivity to drugs, but also influences the penetration of harmful particles inhaled through the respiratory tract into the body. Therefore, this factor must be considered when designing CF OrgOC models.

#### 3.1.2 Dynamic fluid perfusion

Organoids are three-dimensional miniature organ models formed through the self-organization of stem cells, and their maturation—encompassing structural integrity, functional differentiation, and simulation of physiological characteristics—depends heavily on the optimization of the culture environment. Uninterrupted dynamic fluid perfusion simulates the continuous physiological flow environment of in vivo organs through three key mechanisms: stabilizing the microenvironment (mitigating nutrient and metabolic fluctuations), continuously removing waste (preventing toxic accumulation), and providing sustained mechanical signals (promoting functional differentiation). This facilitates the 'progressive maturation' of organoids, transitioning from 'cell clusters' to 'functional miniature organs.' Tao et al. employed an OrgOC platform to create heterogeneous human pancreatic islet organoids from human induced pluripotent stem cells (hiPSCs) [131]. The device comprises four components: the top and bottom PDMS layers are separated by through-hole PDMS membranes and polycarbonate porous membranes (Fig. 5B). The microporous array in the top layer is created by a through-hole PDMS membrane and a polycarbonate porous membrane, facilitating 3D culture of embryoid bodies (EBs) and continuous medium perfusion. One of the polycarbonate porous membranes is used to interconnect media flow between the upper and lower channels of the device. The multilayer chip design enables recirculation of the flow, thereby ensuring adequate media exchange and uniform fluid stress on the islet-like organoids. The heterogeneous islet-like organoids formed under these culture conditions exhibited enhanced expression of pancreatic β-cell-specific genes and proteins (PDX1 and NKX6.1), as well as increased expression of β-cell hormone-specific INS genes and C-peptide proteins, compared to static cultures. These results demonstrated the promotive role of bionic mechanical flow in the differentiation and maturation of endocrine cells in pancreatic islet organoids. Traditional static-cultured CF organoids (e.g., airway organoids) often exhibit phenotypic 'drift' (e.g., transient decrease in mucus viscosity following medium changes, followed by a rebound due to metabolic waste accumulation) due to environmental fluctuations (e.g., osmotic pressure changes caused by medium replacement) and the absence of mechanical stimulation, failing to consistently simulate CF pathological progression. Continuous dynamic fluid perfusion, by mimicking the 'continuous flow' microenvironment of the airways, can rectify these defects and ensure the stable expression of CF phenotypes.

### 3.2 Vascularization of organoids

As organoids increase in size, diffusive transport becomes inadequate to meet their growing metabolic demands and ultimately fails to support their growth and maturation [132]. Vascularization of organoids represents an emerging strategy to overcome the challenges of limited nutrient availability and sustain longevity in traditional organoid models. Consequently, OrgOC are being developed to replicate microvessel formation and vascularization within organoids.

#### 3.2.1 Vasculogenesis

**Fig. 6:**
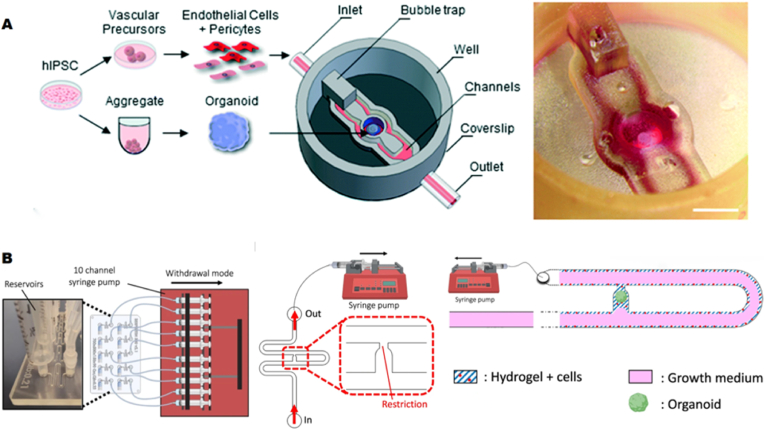
**Construction of microvascular organoids-on-a-chip** (A) Assembly process of microvascularized organoid-on-a-chip: Human pluripotent stem cells (hPSCs) were differentiated into vascular cells and early neural organoids in suspension, and subsequently inoculated into the 3D-printed microfluidic chip. Physical diagram of the microvascularized organoid-on-a-chip platform. Reproduced with permission [133] Copyright 2022, The Royal Society of Chemistry. (B) Diagram of a high-throughput microvascularized organoid-on-a-chip system with ten microchannels, each controlled simultaneously by syringe pumps. Intra-channel structure featuring hydrogel containing endothelial cells attached to the channel walls, organoids captured in traps, and culture medium perfused through the channels via syringe pump for continuous flow. Reproduced with permission [136]. Copyright 2024 Springer Nature.

Vasculogenesis refers to the process in early embryonic development wherein undifferentiated endothelial progenitor cells aggregate, differentiate, and form a primitive vascular network. Salmon et al. developed a 3D-printed microfluidic organoid chip featuring a central 'open' organoid culture chamber flanked by microfluidic channels and a bottom coverslip, enabling precise spatial control and targeted growth of vascular cells (Fig. 6A) [133]. The generation and co-culture of vascularized organoids were achieved by differentiating human pluripotent stem cells (hPSCs) into vascular cells (endothelial cells and pericytes) and early neural organoids, which were subsequently inoculated into the 3D-printed microfluidic chip. By co-culturing these differentiated vascular cells and organoids in the microchip, the researchers observed the spontaneous formation of an orderly vascular network around the organoids. Moreover, the permeability and integrity of these vascular networks were verified through functional assays.

#### 3.2.2 Angiogenesis

Angiogenesis refers to the process by which new blood vessels are formed from pre-existing vessels through the proliferation, migration, and sprouting (or bifurcation) of endothelial cells. The intrinsic bio-programming within endothelial cells (ECs) and stromal cells facilitates the self-assembly of the vascular network. By simply placing ECs and stromal cells together in a hydrogel (e.g., collagen, fibronectin, or matrix gel), the ECs migrate autonomously, make contact, align, form a lumen, and generate an intact, perfusable network [134,135]. For instance, Quintard et al. developed a microvascularized OrgOC platform that facilitated and monitored the formation of vascular endothelial networks (Fig. 6B) [136]. Each chip contained 10 microchannels and was monitored using a 10-channel syringe pump for precise control. The channels were designed as serpentine microchannels with a central culture chamber, which allowed for precise localization of the organoid. The channel walls were lined with a fibrin hydrogel containing human umbilical vein endothelial cells (HUVECs) and fibroblasts, enabling the formation of a self-organizing endothelial network that traversed the trap site, thereby providing nutrients to the organoids. The microarray platform was capable of culturing vascular and islet organoids derived from (hiPSCs for up to 30 days.

Maintaining the growth and extending the lifespan of organoids are fundamental prerequisites for their application in chronic disease modeling. One of the core pathologies in the airways of CF patients is chronic inflammation, characterized by the continuous infiltration of immune cells (e.g., neutrophils and macrophages) through blood vessels, which release inflammatory factors and toxic substances, leading to an "imbalance between injury and repair." Avascular organoids lack a vascular network, which limits immune cells' interaction with organoids to static co-culture in a "non-physiological contact" manner, thereby failing to simulate the dynamic "circulation-adhesion-extravasation" process in vivo. In contrast, vascularized CF OrgOC can accurately replicate this critical pathology through their vascular network.

## 4 Discussion and conclusions

Organoids and OOC technologies have made significant strides in recent years, particularly in constructing CF disease models, advancing drug screening, and facilitating mechanistic studies. Organoids derived from CF patient bronchial or rectal epithelial cells (e.g., airway and rectal organoids) have successfully recapitulated key pathological features associated with CFTR mutations, including abnormal mucus secretion, ciliary dysfunction, and chronic inflammation. Moreover, patient-derived organoids allow for direct assessment of CFTR channel function through FIS assays, enabling both accurate CF diagnosis and personalized drug screening for the disease. In the realm of OOC models, human lung airway chips simulate the dynamic airway microenvironment (e.g., mucus flow shear stress, mechanical stress) using microfluidic technology. This approach enables a more precise replication of structural features (e.g., ciliated cell coverage, mucin accumulation) and inflammatory responses (e.g., increased secretion of pro-inflammatory cytokines such as IL-8, immune cell infiltration) observed in CF patients. Additionally, by inoculating *Pseudomonas aeruginosa*, these models can further replicate the immune response following lung infection in CF patients, including polymorphonuclear leukocyte recruitment and inflammatory cytokine release. Consequently, this platform provides a dynamic and versatile tool for infection mechanism studies and the evaluation of antibacterial drug efficacy.

Despite the significant advances in organoid andOOC technologies, their clinical translation is still hindered by several bottlenecks. In organoids, substantial batch-to-batch variability in both structure and function persists, compounded by the lack of vascular networks and incomplete tissue complexity. This results in insufficient long-term culture capabilities and a limited ability to simulate complex pathological processes. Furthermore, issues such as reliance on static culture media for nutrient supply and the low standardization of experimental procedures undermine data reliability and reproducibility. OOC technology, on the other hand, faces dual challenges: technical complexity and high costs. The fabrication and operation of high-precision microfluidic chips require specialized equipment, while the development of multi-organ linkage chips remains in the early stages, making it difficult to fully replicate in vivo multi-organ interactions. Moreover, variability in chip design and culture conditions across different laboratories impedes the comparability of results, and the simulation of key pathological features, such as chronic inflammation, remains suboptimal.

As an emerging interdisciplinary field that integrates organoid and OOC technologies, OrgOC remains in the early exploratory stages of CF research, yet it holds considerable potential. The future development of OrgOC technology in CF pathophysiology and precision therapy can be directed toward multiple key areas, including technological optimization, multidisciplinary integration, standardization, and the facilitation of clinical translation. From a technological perspective, given the monogenic nature of CF, characterized by CFTR channel dysfunction, it is crucial to improve the fidelity of organoids and disease models. This can be achieved through the advanced integration of 3D bioprinting and microfluidic technologies, which would allow for precise control over cell seeding density and extracellular matrix composition. Such integration would enable the construction of organoid structures that more accurately replicate target organs of CF patients, such as the airways and pancreas, while also simulating critical pathophysiological features, including abnormal mucus secretion and ciliary dysfunction.

Additionally, the incorporation of functional vascular networks (e.g., endothelial cell co-culture) and the dynamic regulation of the microenvironment (e.g., microfluidic perfusion to simulate mucus flow shear stress, mechanical stress loading to replicate cough-induced pressure changes) can significantly enhance nutrient delivery and metabolic waste clearance in organoids. This approach overcomes several limitations of traditional organoid models, including size constraints and the inadequate simulation of complex pathological processes.

In the context of multidisciplinary integration, the convergence of artificial intelligence (AI) and big data technologies is poised to significantly accelerate drug screening and mechanistic research. By analyzing real-time monitoring data from OrgOC models—such as CFTR channel opening frequency and dynamic changes in mucus viscosity—AI can rapidly identify biomarkers associated with drug sensitivity and resistance to CFTR modulators. The integration of patient genomic data further enables the development of a "genotype-organoid phenotype-drug response" predictive model, thereby enhancing the precision of personalized medicine. Moreover, the advancement of multi-organ linkage OrgOC platforms, which simulate multi-organ damage resulting from CFTR dysfunction (such as pancreatic exocrine insufficiency and cholestatic liver disease), can address the limitations of single-organ models. These integrated systems provide a more physiologically relevant platform for studying systemic pathology, drug metabolism, and toxicity, thus offering a comprehensive tool for therapeutic evaluation.

Standardization and clinical translation are critical steps in advancing the implementation of OrgOC technologies. For CF OrgOC systems, it is essential to establish both global and domestic technical validation standards (e.g., organoid differentiation efficiency, CFTR functional assay protocols) and to develop clinical-grade, animal-free culture media, along with biocompatible chip materials [137] exhibiting low drug adsorption. This would minimize batch-to-batch variability and enhance data reliability. In expanding the range of application scenarios, patient-derived OrgOC models facilitate rapid CFTR modulator sensitivity testing, offering precise medication guidance for patients with rare CFTR mutations. These models can also replace certain animal experiments, thereby reducing drug development cycles and significantly lowering R&D costs. Moreover, leveraging OrgOC technology to model CFTR-related defects in immune cell recruitment and activation enables a deeper exploration of chronic infection mechanisms in CF patients, presenting novel targets for anti-inflammatory and immunomodulatory drug development.

With the ongoing advancement and validation of OrgOC technology, it is poised to play a pivotal role in enhancing our understanding of various disease mechanisms and in guiding the development of novel therapeutic strategies. Consequently, it is anticipated that OrgOC technology will enable the provision of more precise and effective personalized treatment options for CF patients, thereby significantly improving their quality of life and extending their lifespan.

## CRediT authorship contribution statement

**Minjie Zheng:** Writing – review & editing, Writing – original draft, Conceptualization. **Elisa Erice:** Writing – review & editing, Writing – original draft, Supervision, Conceptualization. **Huiyi Wang:** Writing – review & editing, Writing – original draft, Supervision, Conceptualization. **Lei Zhang:** Writing – review & editing, Writing – original draft, Supervision, Project administration, Funding acquisition, Conceptualization. **Charles H. Lawrie:** Writing – review & editing, Writing – original draft, Resources, Project administration, Funding acquisition, Conceptualization.

## Declaration of competing interest

The authors declare that they have no known competing financial interests or personal relationships that could have appeared to influence the work reported in this paper.

## Data Availability

No data was used for the research described in the article.
